# Quality of life to four years in traumatic brain injury critical patients

**DOI:** 10.1186/2197-425X-3-S1-A375

**Published:** 2015-10-01

**Authors:** MD Arias-Verdu, E Aguilar-Alonso, G Jimenez-Perez, E Curiel-Balsera, M Delange-Van Der Kroff, A Muñoz-López, E Moran-Fernandez, JF Fernández-Ortega, R Rivera-Fernandez, MA Prieto-Palomino

**Affiliations:** Intensive Care, Regional University Hospital, Malaga, Spain; Intensive Care, Infanta Margarita Hospital, Cabra, Spain; Intensive Care, Axarquía Hospital, Velez-Malaga, Spain

## Introduction

Many instruments have been developed to evaluate hospital mortality, but less attention has been paid to the long-term functional status and quality of life of traumatic brain injury patients.

## Objectives

To analyze the quality of life after four years in traumatic brain injury critical patients admitted to intensive care.

## Methods

Prospective cohort study of traumatic brain injury patients admitted in the University Clinical Hospital (Malaga) between 2004 to 2008.

## Results

531 patients. Mean age 40.35 ± 19.75 years, APACHE-II 17.94 ± 6.97, admission GCS 7.53 ± 3.83 points. Computerized tomography (CT) on admission by Marshall score was: diffuse injury type I (10.4%), type II (28.1%), type III (24.5%), type IV (8.3 %), mass evacuated (22.6%), mass not evacuated (6.2%). Hospital mortality 28.6%. 171 patients died at first year (32.2%) (Lost 6.6%) and 181 at 4 years (34.1%) (Lost: 16.2%).

The evaluation of the quality of life was performed by PAECC (Project for the Epidemiological Analysis of Critical Care Patients) QOL (Quality of Life) questionnaire (0 points: normal quality of life, 29 points: worst score). Mean score at first year follow-up 9.44 ± 8.73 points (N = 324), indicating high deterioration in quality of life. Mean score at 4 years follow-up was 6.77 ± 7.70 points (N = 238), indicating moderate deterioration in quality of life (p < 0.001).

There is an association between between quality of life for four years with the tomographic Marshall score. Quality of life at 4 years in patients with diffuse injury type I was 4.12 ± 5.27 points, with type II 4.91 ± 5.27, 9.05 ± 8.52 with type III, with type IV 13.71 ± 9.51, in evacuated mass 7.11 ± 9 (p < 0.001).

Multivariate analysis found association between quality of life for four years with the tomographic Marshall score, the depth of coma by GCS, age, hospital stay and functional status by Glasgow Outcome Scale. There was no statistically significant relationship with sex, spinal cord injury, APACHE II and Injury Severity Score (ISS).

## Conclusions

Traumatic brain injury patients have at four years a high rate of mortality and poor quality of life. The quality of life for four years has important relationship with cranial CT on admission.
